# ACT-related Processes: Value-based Living Attenuates Meditating Relationships in Caregiver Stress Outcomes

**DOI:** 10.1093/geroni/igab046.2975

**Published:** 2021-12-17

**Authors:** Elizabeth Braungart Fauth, Joshua Novak, Ty Aller, Heather Kelley, Michael Levin

**Affiliations:** 1 Utah State University, Logan, Utah, United States; 2 Auburn University, Auburn, Alabama, United States

## Abstract

Associations between behavioral and psychological symptoms of dementia (BPSD), caregiver burden, and depressive symptoms are well-established, and these constructs are often targeted in interventions. Increasingly, dementia caregiver interventions are informed by mindfulness- and acceptance-based approaches, such as Acceptance and Commitment Therapy (ACT). In addition to standard outcomes, like burden and depressive symptoms, these interventions/therapies seek improvements in individuals’ psychological flexibility (e.g., cognitive defusion, present moment awareness, values-based living). Less is known how these constructs interact within well-established caregiver stress processes. We examined a moderated mediation model (N=161 dementia caregivers; PROCESS Procedure; SPSS Release 2.16.1), with BPSD frequency (Revised Memory and Behavior Problems Checklist) predicting depressive symptoms (10-item CES-D), mediated via caregiver burden (short Burden inventory). The moderator was the Values Questionnaire (Progress scale), and we controlled for gender, caregiver duration, age, income, and education. Results revealed that the indirect effect of BPSD on depressive symptoms through caregiver burden was weakened through higher progress toward values (moderated mediation significant at p<.05). In essence, greater levels of living according to values dampened the effect of BPSD on depressive symptoms, through care-related burden. These findings are important because caregivers often cannot leave this role, requiring them to learn to live with the caregiving role in healthy ways. Value-based living and committed action toward values signify caregivers’ success at balancing care-related stress with other priorities, and psychologically adjusting to difficulties. Interventions that emphasize values-based living, like ACT, have promise for caregivers, offering healthy ways to psychologically adjust to, and live with, the experience.

